# Beyond the Safe Motherhood Initiative: Accelerated Action Urgently Needed to End Preventable Maternal Mortality

**DOI:** 10.9745/GHSP-D-18-00100

**Published:** 2018-10-03

**Authors:** Mary Ellen Stanton, Barbara E. Kwast, Theresa Shaver, Betsy McCallon, Marge Koblinsky

**Affiliations:** a United States Agency for International Development, Washington, DC, USA.; b Independent consultant, Leusden, The Netherlands.; c White Ribbon Alliance, Washington, DC, USA.; d Independent consultant, Washington, DC, USA.

## Abstract

Many countries will need to double, or more than double, their current annual rate of reduction of maternal mortality to ensure sufficient progress toward national targets and the global Sustainable Development Goals. Dedication to the principles and actions of quality, equity, dignity, social justice, and human rights are key.

Following the 30th anniversary of the launch of the global Safe Motherhood Initiative, the world now looks ahead to renewing its visionary goal of ending preventable maternal mortality. Progress toward that aspiration is often equated with the 44% reduction in global maternal mortality between 1990 and 2015.^1^ Yet this reduction was still far from achieving Millennium Development Goal (MDG) 5 of reducing the maternal mortality ratio (MMR) by three-quarters between 1990 and 2015, and it is even farther from achieving Sustainable Development Goal (SDG) 3, which aims to attain a global MMR of less than 70 deaths per 100,000 live births by 2030.

Today there are more than 300,000 maternal deaths each year worldwide.^1^ More than 200 million women wish to avoid, delay, or end childbearing but are not using contraception.^2^ The face of these numbers is increasingly an adolescent girl—complications during pregnancy and childbirth are the leading cause of death of 15 to 19-year-old girls globally.^3^

While facility delivery is rapidly increasing, even now more than 30 million women deliver yearly without the care of a skilled birth attendant.^4^ Many, including those who do deliver in hospitals, receive substandard care in inadequate facilities and face disrespect and abuse from exhausted health care workers who are often disrespected themselves. Most significant of all is the unconscionable disparity between rich and poor nations—the lifetime risk of maternal death is more than 100 times greater in sub-Saharan Africa than in Europe. Furthermore, within nations there are inequities between subpopulations. Among the multitude of determinants, conflict, poverty, inequity, and unrealized political promises are significant factors. And, while this article focuses on maternal mortality, we would be remiss not to mention the significant burden of maternal morbidity and disability, as well as the 2.6 million newborns who die every year and an equal number who are stillborn.^5^

The overall MMR annual rate of reduction needed to reach the SDG target for maternal mortality reduction is −7.5%, significantly higher than the −5.5% needed to meet MDG 5.^6^ It is sobering to note that the annual rate of reduction attained globally was only −3.0% between 2000 and 2015. Across the globe, progress in maternal mortality reduction within and between countries is highly variable. In some countries progress is stalling, with annual rates of reduction between 2005 and 2015 slowing in relation to earlier rates.^1^ Even maintaining the current positive momentum in other countries will not be sufficient to meet the global SDG for maternal mortality reduction. Although there are limitations to drawing conclusions from point estimates of maternal mortality, apparent slowing or stalling in some countries should be treated as an alert—analyzed and urgently addressed.

A −7.5% annual rate of reduction is needed to reach the SDG target for maternal mortality reduction, significantly higher than the −5.5% needed to meet the MDGs.

At this point, substantially accelerated and continuous progress requiring additional investment is urgently needed across most countries. Yet after 2013, there has been a leveling out of development assistance for health funds for maternal health.^7^

## THE CHANGING SCENE AFFECTING MATERNAL HEALTH

Global understanding of the determinants of maternal mortality and a global vision of ending preventable maternal mortality have resulted in a shift in global efforts from the “Safe Motherhood Initiative” launched in 1987 in Nairobi to “Ending Preventable Maternal Mortality” in 2014 in Bangkok.

Over the decades, technical and programmatic achievements included attention to the major direct causes of maternal mortality, the competencies of the skilled birth attendant, the place of birth, and the enabling environment. The focus widened as morbidity became more visible, initially to include obstetric fistula, and then to other morbidities and disabilities.

Programming approaches integrated infectious disease control, especially HIV and malaria, and family planning. Furthermore, the strengthening of health systems to ensure communication and the means of referral within and between levels of care and to ensure quality of care was identified as indispensable. Indeed, maternal survival has been identified as a bellwether for strengthened health systems and inter-sectoral collaboration.

There was growing recognition that maternal and perinatal health are inextricably linked. Gradually programmatic approaches became oriented to both maternal and newborn care. Actions taken to achieve Ending Preventable Maternal Mortality and Every Newborn Action Plan goals are being conducted in close coordination.

The global Ending Preventable Maternal Mortality strategy^8^ moves beyond emphasis on clinical care to address the social, political, and economic determinants of maternal survival and health that have contributed to countries' failures and successes.

Significantly, maternal biomedical interventions, community approaches, and financial incentives, to name just a few, have been subjected to scientific inquiry, from qualitative studies to randomized control trials to implementation research. The lack of evidence that characterized obstetrics and midwifery since the beginning of these disciplines is no longer the norm. In recent years, expert groups led by the World Health Organization (WHO) representing all regions of the world have scrutinized increasingly available studies and formulated evidence-based recommendations and guidelines for normal care and complications, community mobilization, and health promotion.

The *Maternal Health Lancet Series 2016*,^9^ following the first series from 2006, included analyses of drivers and external shocks that influence progress. “Too little, too late” and “too much, too soon” characterize the poor quality of care and the rapidly increasing, inappropriate medicalization of care.

“Too little, too late” and “too much, too soon” characterize the poor quality of care and the rapidly increasing, inappropriate medicalization of care.

Until recently, maternal health commodities were almost entirely neglected. The UN Commission on Life-Saving Commodities for Women and Children brought international attention at the highest levels and galvanized attention to production, supply, and use of affordable, high-quality products. Increasingly at national levels, there is testing of drugs for potency and attention to stock-outs and proper use, with initial attention to uterotonics, anticonvulsives, and antibiotics.

During the past decade, attention has gone beyond statistics and biomedical interventions, to focus on women themselves and their experience of maternity care. Qualitative and quantitative research and media reports have exposed disrespect and abuse of women in childbirth in health facilities that includes physical abuse; lack of privacy, confidentiality, and consent; non-dignified care including verbal abuse; neglect and even abandonment or detention in facilities; and lack of resources in the facilities including bed space, light, water, and sanitation.^10^^,^^11^ Such mistreatment is increasingly recognized as poor quality of care and one of the biggest barriers to women seeking care.

The issue of respect has gained traction with listening to women now recognized as necessary. Human rights and social justice issues were highlighted in the White Ribbon Alliance's 2011 charter, *Respectful Maternity Care: The Universal Rights of Childbearing Women,*^12^ and in WHO's 2014 statement, *The Prevention and Elimination of Disrespect and Abuse During Facility-Based Childbirth*.^13^ The right of women to determine where and with whom they will give birth and to have a companion of choice is critical. Recently, WHO guidelines for antenatal and intrapartum care have specified guidance for a positive pregnancy and birth experience. Also, WHO standards for improving quality of maternal and newborn care in health facilities delineates both the experience and the provision of care as part of a forward-thinking framework for quality of care.

There is growing understanding that working conditions for midwives and other health workers—including low pay, excruciatingly long hours, inadequate supplies, and being mistreated themselves—can contribute to poor treatment of women.^14^ Nations are increasingly funding their own health programs as external support diminishes and becomes a smaller proportion of overall costs. Yet still of concern are the considerable out-of-pocket costs that can impoverish families and lead to death of mothers or their newborns.

## CONTEXT MATTERS

Since the 1990s, national successes in maternal mortality reduction have ridden the waves of economic growth, translating such growth into better infrastructure, referral systems, and care. Conversely, conflict, natural disasters, and infectious disease outbreaks have created additional vulnerabilities for childbearing women. Population migrations and the recent Zika and Ebola outbreaks have highlighted the reality of these concerns.

Societal, technologic, and political shifts, as well as well-intentioned interventions, may result in variable outcomes for maternal health and survival in the coming decade:

- Growing international attention to non-communicable diseases brings important linkages with hypertension, obesity, and diabetes to maternal health programs to avert many direct and indirect causes of maternal ill health. Likewise, attention to water, sanitation, and hygiene (WASH) is critical. Nevertheless, we must recognize that this growing agenda in public health may redirect attention from the unfinished agenda of maternal and child survival.
- The use of financial incentives for women and health care providers has provided the impetus to rapidly increase coverage of care in health facilities for birth. This has led, in some places, to women crowding into inadequate facilities with under-resourced services, supplies, and personnel, where even the basics of quality, respectful care are not available. In many countries, improved use of facility care for birth has not been linked with improved outcomes—signaling greater need for investment in quality improvement.
- Decentralization brings opportunity to local leaders and structures to determine their own priorities and ensure accountability. It also demands capacities to plan, budget, implement, and monitor programs sub-nationally. Building these competencies is not uniformly rapid, even as it provides great opportunities when excellent leadership is in place.
- Urbanization brings many closer to health care services, but slums and the complexity of urban environments may subject childbearing women and their babies to infectious diseases, poor sanitation, and stress of a different kind than experienced in a home village.
- The expansion of private-sector services has improved access for many, but without consistent and enforced regulations and standards, quality varies dramatically and accountability is too often lacking. Companies that are developing new markets for high-cost equipment can create demand and draw on budget allocations in places where there is insufficient basic equipment to detect and treat common life-threatening complications.
- As training in global standards and vital skills that save the lives of women and newborns is a growing priority for nurses, doctors, and midwives, newly trained staff face constant rotation within facilities and countries, denying them the ability to retain these skills effectively. Furthermore, once trained, health workers may become more attractive to private facilities or foreign countries who can pay more, contributing to the challenge of retaining a skilled health workforce.
- Innovations provide great promise as we tackle the problems of finding better, cheaper diagnostics and treatments, and using communication technologies to their full advantage. At the same time, known effective approaches or systems improvements may be overlooked as we look for “silver bullets.”
- Mobile technologies and social media have expanded messaging to and communication with consumers of care and diminished the isolation of rural centers of care. However, the improved messages to families may drive increased expectations and use of services before services are ready and may incur considerable cost without evidence of improvement in outcomes or health impact.
- Economic motivation can be galvanizing to create services but may also be detrimental to women's care and health. Expectation of informal payments—even in environments where there is a “free” maternity care policy—can result in poor quality or delay or abandonment of women in urgent need.
- While there has been increased attention to the issue of adolescent pregnancy, a multi-sectoral approach is also required to tackle restrictive laws and policies, health worker bias, social and cultural support for child marriage, and limited knowledge and support for decision making by adolescents themselves.
- The increasing medicalization of pregnancy and birth has expanded access for some women to lifesaving care while also subjecting other women to unnecessary medication and surgery, including induction and augmentation of labor and cesarean delivery, resulting in such complications as uterine rupture, infections, iatrogenic fistula, hemorrhage in subsequent pregnancies, and even death. In addition, the medicalized environment can result in an alienating environment for women during labor and birth and unnecessary costs to the health system.

## RENEWING THE COMMITMENT

The SDGs for 2030 include an ambitious target for maternal mortality reduction in just 12 years. In the face of stalled progress in some countries and an inadequate rate of progress in all but a few countries, many countries will need to double, or more than double, their current annual rate of reduction of maternal mortality to ensure sufficient progress toward national targets and contribution to the global SDG and universal health care.

In the face of stalled progress, many countries will need to double, or more than double, their current annual rate of reduction of maternal mortality to ensure sufficient progress toward national and global targets.

Each nation will need to set its own course with a rededication to the “unfinished agenda” of ending preventable maternal mortality with context-specific actions. These include effective policies, adequate budgets, and state-of-the-art monitoring and quality improvement systems.

The Global Financing Facility, is an important “country-powered” effort to reduce the massive funding gap for reproductive, maternal, newborn, child, and adolescent health and nutrition programs. ^15^ The promise of new economic resources, partnerships and innovation is welcome. We must put these assets to good use.

There must be a dramatic paradigm shift so that women are valued as drivers of their own health, not mere passive recipients of care. Dedication to the principles and actions of quality, equity, dignity, social justice, and human rights are key to catalyzing additional and vital action toward our vision: **No woman, no matter in what country she resides, should die of complications during pregnancy, childbirth or the postpartum period, nor lose her newborn in the process.**

Success will require that ending preventable mortality remains a political priority.^16^ This will necessitate strengthening of networks, individuals, and organizations to keep up the pressure, push for investment in what works, be bold in tackling new challenges, and ultimately support nations to have resilient health systems to meet the needs of all people.

## Notes

**Peer Reviewed**

**Funding:** We have no funding to declare for preparation of this article.

**First Published Online:** August 8, 2018
